# Value of Character

**Published:** 1845-03

**Authors:** 


*Value of Character.*—Colonel Chartres, who was the most notorious rascal in the world, and who had, by all sorts of crimes, amassed immense wealth, sensible of the disadvantages of a bad character, was once heard to say, that "although he would not give one farthing for virtue, he would give ten thousand pounds for a character, because he should get a hundred thousand pounds by it." Is it possible then, that an honest man can neglect what a wise man would purchase so dear?—*Lord Chesterfield.*

